# A lightweight hybrid CNN and transformer model for medicinal leaf disease classification with explainable AI

**DOI:** 10.1038/s41598-026-39182-3

**Published:** 2026-02-11

**Authors:** Jalal Ahmmed, Md Alamgir Kabir, Atiq ur Rehman, Amine Bermak

**Affiliations:** 1 Department of Computer Science and Engineering, Daffodil International University, Dhaka, Bangladesh; 2 College of Science and Engineering, Hamad Bin Khalifa University, Doha, Qatar

**Keywords:** Medicinal plants, Leaf disease classification, Hybrid CNN–transformer, Explainable AI, Precision agriculture, Computational biology and bioinformatics, Plant sciences

## Abstract

Medicinal plants including *Ocimum tenuiflorum L.* (Tulsi), *Azadirachta indica A. Juss.* (Neem), and *Kalanchoe pinnata (Lam.) Pers.* (Patharkuchi) are essential sources of bioactive compounds, yet leaf diseases threaten their yield and phytochemical integrity. This study proposes LSeTNet, a lightweight hybrid CNN (Convolutional Neural Network) Transformer architecture with Squeeze-and-Excitation (SE) blocks, achieving 99.72% accuracy, 1.00 macro F1-score, and AUC = 1.00 across 12 disease classes (1,000 images/class post-augmentation) using only 9.38 M parameters and 2.50 GFLOPs. Five-fold cross-validation yielded 99.74% ± 0.14% accuracy, with rapid convergence and no overfitting. Explainable Artificial Intelligence (XAI) via Gradient-weighted Class Activation Mapping (Grad-CAM) (mean intensity: 0.1664–0.2702), Local Interpretable Model-agnostic Explanations (LIME), and t-distributed Stochastic Neighbor Embedding (t-SNE) (silhouette score: 0.87) confirmed biologically meaningful attention on pathological regions. External validation on the independent BD-MediLeaves dataset (8 classes, 8,000 samples) achieved 99.42% accuracy and 0.99 macro F1. With 6.98 ms/image inference latency and 35.81 MB memory, LSeTNet enables real-time, edge-based deployment. It significantly outperforms DenseNet169 (95.56%), ViT-B16 (95.61%), and LW-CNN+SE (95.39%) ($p < 10^{-7}$, paired t-tests), establishing a transparent, efficient, and generalizable benchmark for precision phytopathology and sustainable medicinal plant cultivation.

## Introduction

Medicinal plants have long played an important role in human health and have been used as a mainstay of traditional and modern medical systems^1^. The medicinal properties of these plants are due to the bioactive compounds they contain, which help in the treatment of various diseases. As a result, the health and productivity of these plants have become very important for the sustainable development of the pharmaceutical and agricultural sectors^2^. However, the increasing prevalence of plant foliar diseases is significantly hampering the growth and quality of these valuable plants^3^. Among the widely used medicinal plants, *Ocimum tenuiflorum L.* (tulsi), *Azadirachta indica A. Juss.* (neem) and *Kalanchoe pinnata (Lam.) Pers.* (neem) are particularly noteworthy, as they have antibacterial, antiviral, anti-inflammatory and anticancer properties^4^. *Kalanchoe pinnata* helps in wound healing, reduces inflammation, protects the liver and exhibits anti-cancer activity^5^. *Azadirachta indica* acts as an antibacterial, purifies the blood, boosts immunity and helps in preventing cancer cells^6^. *Ocimum tenuiflorum* boosts immunity, reduces stress, supports respiratory health, and exhibits anti-cancer effects^7^. However, these plants are susceptible to fungal, bacterial, and environmental diseases, which affect yield quality and medicinal efficacy. Therefore, rapid identification and classification of plant foliar diseases is very important to ensure sustainable cultivation and maintain the medicinal potential of these plants^8^.

Traditionally, diseases of medicinal plants are detected by visual observation in the field, which is done by experienced farmers or agricultural experts^9,10^. Although this method is simple and inexpensive, it is highly individual and depends on the experience of the expert. This method is more likely to be wrong in the early stages of the disease or in cases of diseases that are close to appearance. Laboratory and microscopic tests have relatively high diagnostic accuracy and pathogen detection capacity. However, these methods are time-consuming and require special equipment and skilled manpower. Therefore, it is not practical to use these methods on a large scale or in the immediate field. These limitations hinder timely action and make large-scale application difficult. As a result, the need for automated, accurate and interpretable computer-aided disease diagnosis systems becomes clear.

Recent advances in deep learning, especially Convolutional Neural Networks (CNNs) and Vision Transformers (ViTs), have brought about major changes in visual recognition tasks^11^. This includes plant disease detection. CNNs have shown effective performance in feature extraction and pattern recognition from image data. On the other hand, ViT has shown improved performance in capturing long-range spatial relationships through self-attention mechanisms^12^. In the agricultural sector, the use of these models together has enabled more accurate automatic classification of plant diseases. This has reduced the need for manual disease diagnosis by agricultural experts in many cases. However, most existing CNN and ViT-based methods are computationally expensive. These methods are highly dependent on large datasets and often have difficulty generalizing to different plants and diseases^13^.

Recent studies have highlighted several important limitations in the field of disease detection in medicinal plants. Many existing models are developed for a specific plant and fail to generalize to multiple medicinal plants with different leaf structures^14^. Most CNN–ViT based hybrid frameworks are computationally expensive and not suitable for immediate or mobile use^15^. Even when Explainable Artificial Intelligence (XAI) is incorporated, it is often limited to the surface. This limits the explainability and transparency of the model^16^. Furthermore, very few studies have conducted a full evaluation using different backbone frameworks and datasets. Due to this, a clear idea of the robustness and scalability of the model is not available. The existing medicinal plant datasets are also limited or unbalanced. This reduces the reliability of the trained model^17^. More importantly, cross-domain transferability and external validation are rarely tested. This leaves uncertainty about the performance of the model in new species or different environments.^18^. Combined with these limitations, the need for a lightweight, generalizable, and interpretable hybrid framework becomes clear, suitable for real-world disease detection of medicinal plants.

To address the challenge of disease detection in medicinal plants, this study proposes LSeTNet. It is a Lightweight Squeeze-and-Excitation Transformer Network, which combines CNN and ViT^19^. This model effectively balances high prediction accuracy, low computational cost, and good explainability. It uses Explainable AI (XAI) techniques, including LIME^20^ and Grad-CAM^21^. These methods help in clearly identifying the disease location and simplify the decision process. In addition, t-SNE is used to visualize clear differences in class-based traits^22^. This framework uses a diverse dataset including *Ocimum tenuiflorum* (Tulsi), *Azadirachta indica* (Neem), and *Kalanchoe pinnata*. This ensures the robustness and transferability of the model across disease scenarios. Comparisons with state-of-the-art baseline models and external validation on the BD-MediLeaves dataset demonstrate its improved performance. These results present LSeTNet as a scalable solution for precision agriculture applications.

This research makes the following contributions:

1. A multi-class medicinal plant leaf image dataset was curated, covering twelve balanced classes from *Kalanchoe pinnata*, *Azadirachta indica*, and *Ocimum tenuiflorum*. The images were collected under diverse real-field conditions and the dataset is publicly released to support reproducible research.
2. A lightweight hybrid deep learning architecture, named LSeTNet, is proposed by combining convolutional neural networks, Squeeze-and-Excitation blocks, and a Transformer encoder for medicinal leaf disease classification.
3. An explainable AI framework is integrated into the proposed model using Grad-CAM, LIME, and t-SNE to provide visual and feature-level interpretability of model predictions.
4. A systematic evaluation protocol is designed using cross-validation to assess model robustness and stability under different data partitions.
5. The generalization ability of the proposed framework is further examined through evaluation on an independent external medicinal plant dataset.

The remainder of this paper is organized as follows. Section 2 reviews related literature, highlighting advancements in deep learning and explainable AI for plant disease detection. Section 3 details the dataset curation, preprocessing, augmentation, LSeTNet architecture, and XAI methodologies. Section 4 presents the experimental results, which include cross-validation and class-wise performance analysis. This section also presents the interpretability analysis obtained from Grad-CAM, LIME and t-SNE. In addition, comparative analysis including external validation and statistical verification on the BD-MediLeaves dataset is shown. Section 5 discusses the findings, comparing them with prior studies and emphasizing implications for precision agriculture. Section 6 outlines the study’s limitations, conclusions, and directions for future research.

## Related work

Automated medicinal plant identification is gaining much attention due to its importance in pharmaceutical research, biodiversity conservation and traditional medicine. Dey et al.^23^ compared seven CNN models on 5878 images of 30 medicinal plants. In this study, it was found that the deep CNN model is able to effectively capture the fine features of leaves. Kavitha et al.^24^ used MobileNet on six medicinal plants. This clearly shows the advantage of the lightweight model for immediate identification. Panchal et al.^25^ applied transfer learning using InceptionV3, ResNet50 and VGG models on large datasets. This study highlights the importance of model fine-tuning in disease identification.

Hybrid and attention-based networks further improved the identification performance. Kini et al.^26^ Black Paper used InceptionV3, GoogleNet and ResNet18 for disease identification. It also showed good results for nearby disease classes. Pushpa et al.^27^ added Squeeze-and-Excitation blocks to the hybrid CNN framework. It improved the channel-based feature representation in medicinal plant images. Sharma and Vardhan^28^ proposed an AELGNet with local and global attention. Azadnia et al.^29^ presented a spatial–channel attention-based Tree-CA. These studies show that the attention mechanisms focus the model’s attention on disease-related leaf regions rather than the background.

Recent studies have used modified transfer learning frameworks for crop-based disease detection. Lanjewar et al.^30^ modified VGG19, NASNetMobile and DenseNet169 models for potato leaf disease detection. Their method improved the feature discrimination between early and late blight diseases by adding light layers and reducing the trainable parameters. This method relies on a well-designed transfer learning pipeline. Similarly, Panchbhai and Lanjewar^31^ proposed a hybrid framework for tea leaf disease detection. It combined feature selection and machine learning classifiers with a modified modern CNN model. This study shows that using deep and classical learning together increases the robustness of the model.

Multi-task and compact CNN designs have also been studied to improve generalization and efficiency. Sharma and Vardhan^32^ proposed MTJNet, which improves disease classification by learning both vein and edge features simultaneously. These methods demonstrate that compact setups built on task-based constraints can also be effective.

Beyond leaf disease classification, hybrid deep learning models have been applied to agricultural imaging problems. Attri et al.^33^ proposed a quantum-inspired deep learning framework for plant disease detection. Diwedi et al.^34^ combined classical classifiers with CNN feature detection, improving the reliability of the results.

For general leaf disease classification, Talaat et al.^35^ proposed DeepLeaf for grapevine disease detection. Although it is a fuzzy-optimized CNN, it is limited to a single crop and lacks interpretability. Kayaalp^36^ evaluated different deep models on multiple medicinal plants. Binnar and Sharma^37^ demonstrated the effectiveness of MobileNet on large datasets. Despite good results, many studies are limited by individual datasets, limited species, or lack of interpretability.

Table 1 summarizes the methodology, performance, advantages, and limitations of recent important studies. Overall, the literature shows consistent progress in CNN, hybrid, and attention-based models. However, overreliance on transfer learning, limited external validation, and poor interpretability still exist. Single crop-focused studies also remain a common limitation. These shortcomings highlight the need for lightweight and interpretable hybrid deployments applicable to multiple medicinal plants in real environments, which is presented in this study.

**Table 1:** Comparison of recent deep learning approaches for plant/leaf disease classification (selected key works).

Study	Method	Strength	Limitations
Dey et al.^23^	DenseNet201	High accuracy, fine-grained features	Single-crop focus, no XAI, computationally heavy
Kavitha et al.^24^	MobileNet	Lightweight, real-time capable	Limited species coverage
Panchal et al.^25^	Transfer learning (InceptionV3, etc.)	Effective fine-tuning	No interpretability
Kini et al.^26^	Transfer learning (ResNet18, etc.)	Strong on similar classes	Single crop (black pepper), no external validation
Pushpa et al.^27^	Hybrid CNN + SE blocks	Improved channel attention	Limited species, no interpretability
Sharma and Vardhan^28^	AELGNet (local + global attention)	Multi-level attention	No external validation
Azadnia et al.^29^	Tree-CA (spatial–channel attention)	Focused attention on regions	Limited species diversity
Lanjewar et al.^30^	Modified pre-trained models	Lightweight layers	Crop-specific (potato), no XAI
Panchbhai and Lanjewar^31^	Hybrid CNN + ML classifiers	Enhanced robustness	Single crop (tea), limited generalizability
Sharma and Vardhan^32^	MTJNet (vein + edge features)	Multi-task learning	Limited scope
Attri et al.^33^	Quantum-inspired DL	Novel approach	High complexity
Diwedi et al.^34^	CNN + classical classifiers	Improved reliability	No real-time focus
Talaat et al.^35^	DeepLeaf (fuzzy-optimized CNN)	Effective optimization	Single crop, no XAI
Kayaalp^36^	Multiple deep models	Multi-species evaluation	No explainability
Binnar and Sharma^37^	MobileNet	High efficiency	Limited interpretability

## Methodology

**Fig. 1:**
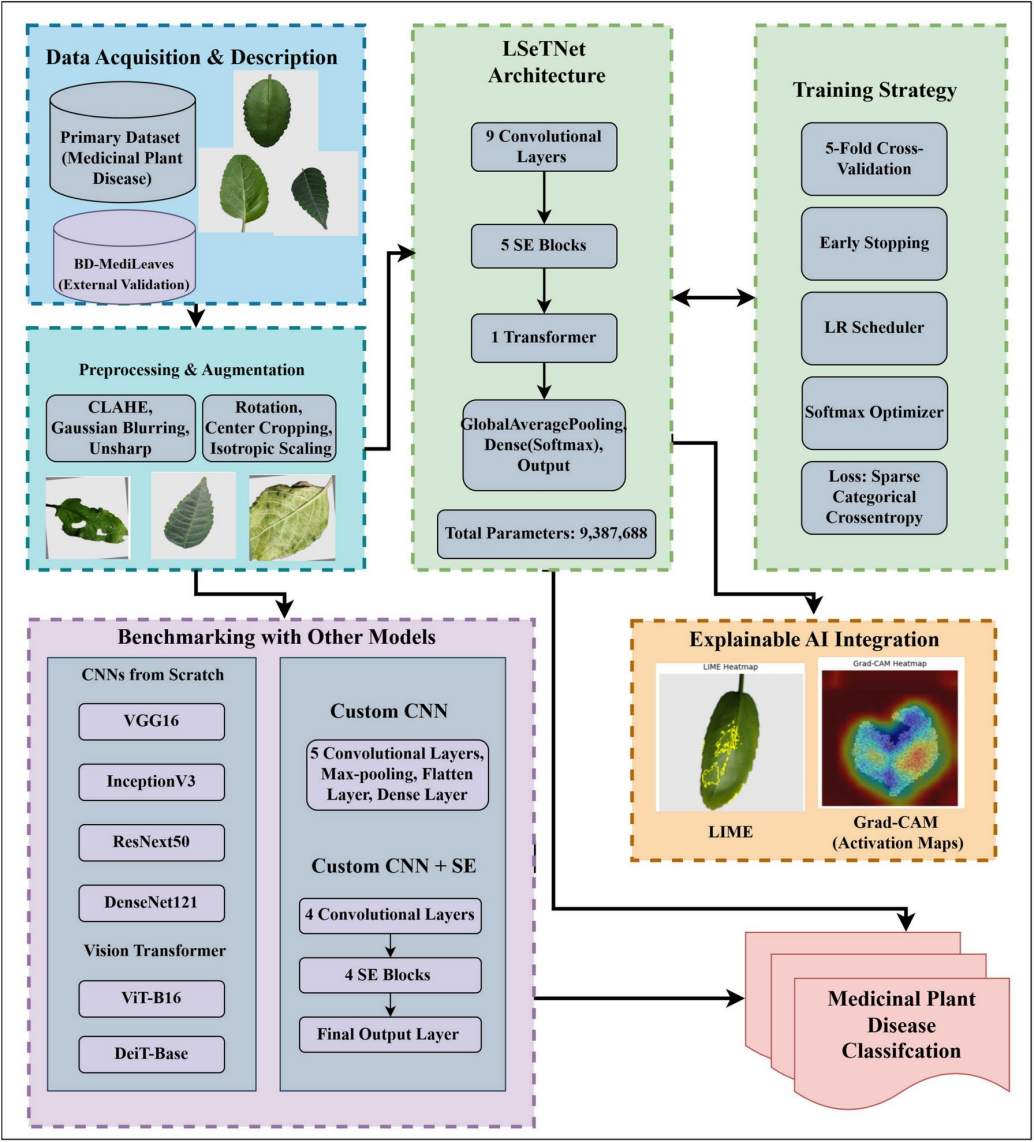
Workflow of the LSeTNet-based methodology, integrating data preparation, model development, benchmarking, and explainable AI for medicinal plant disease classification.

Figure 1 presents the proposed methodology, which includes dataset collection, preprocessing, augmentation, and model development. It also shows the steps of benchmarking and Explainable AI (XAI) integration. The workflow starts with the creation of initial and external validation datasets. Then, image preprocessing and augmentation are applied to increase the robustness of the model. The LSeTNet model is built using a combination of convolutional, Squeeze-and-Excitation (SE), and Transformer layers. The model is trained using cross-validation and adaptively optimized. The performance is evaluated by comparing it with baseline and custom deployments. LIME and Grad-CAM visualization are used to enhance explainability. The framework ensures reliable and explainable disease classification of medicinal plants. The results are validated through statistical analysis.

### Dataset description

The initial dataset includes high-resolution images of medicinal plant leaves. These images were collected in August 2025 in Senbagh area of Noakhali, Bangladesh. The dataset contains three plant species. These are *Kalanchoe pinnata* (Lam.) Pers. (patharkuchi), *Azadirachta indica* A. Juss. (neem) and *Ocimum tenuiflorum* L. (tulsi). For each species, one healthy class and three disease or symptom classes are maintained. This results in a total of twelve balanced classes. Each class contains 200–225 original images. The total number of samples is approximately $\sim$2547.

The disease classes are determined based on visible symptoms according to established plant pathology guidelines^9,10^. All images were captured in natural field environments. Different angles, lighting and backgrounds are included. The aim is to reflect the diversity of real environments and increase the robustness of the model. The class labels are determined based on field-level symptoms. These were verified by the Agriculture Officer of Senbag Upazila on 19 October 2025. The verification process included direct field visits, expert advice and confirmation of local disease prevalence.

The classes of *Kalanchoe pinnata (Patharkuchi)* are:

- *Healthy* Even green leaves, edges intact and no discoloration.
- *Web Blight* Gray mycelial web and confluent necrotic spots, caused by Rhizoctonia-like fungal infection^9^.
- *Yellow* Yellow color throughout the leaf, but veins remain green, which usually indicates nutrient deficiency or initial viral stress.
- *Yellow Blight* Extensive yellowing followed by marginal necrosis and tissue destruction. This is a syndrome observed in the field in the area.

The classes of *Azadirachta indica (neem)* are:

- *Healthy* Deep green leaves, no spots or discoloration.
- *Leaf Spot* Clear brown spots with a circular ring, which are common in fungal leaf spot diseases^10^.
- *Web Blight* Silky fungal webs that cause leaf curling and necrosis.
- *Yellow* General yellowing and drooping of leaves, which is a sign of nutritional stress or early systemic infection.

The classes of *Ocimum tenuiflorum (tulsi)* are:

- *Healthy*: Fresh green leaves, no spots or discoloration.
- *Downy Mildew*: Angular yellow spots and a gray-purple coating on the underside of the leaves, characteristic of Peronospora infection^9^.
- *Web Blight*: Thin fungal web on the leaves, causing necrosis and tissue death.
- *Yellow Spot*: Light yellow ring around the initial lesion, common in bacterial or fungal spot diseases.

**Fig. 2:**
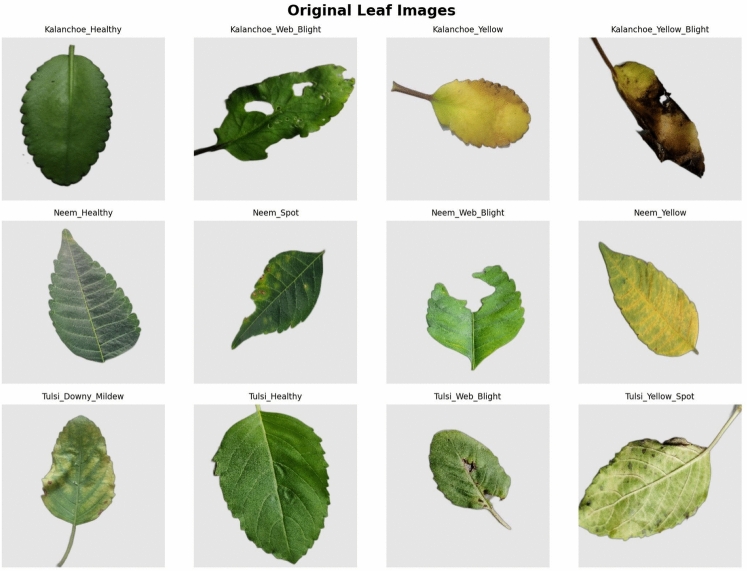
Sample images from the MedicinalLeaf-12 dataset, showcasing healthy and diseased leaves of *Kalanchoe pinnata*, *Azadirachta indica*, and *Ocimum tenuiflorum*. The Kalanchoe_Yellow_Blight class (top row, far right) displays combined chlorosis and marginal blight.

This dataset accurately represents the field symptoms common in the coastal regions of Bangladesh. It includes fungal, bacterial and environmental stress symptoms. The dataset is published in Mendeley Data for complete transparency and reproducibility^38^. Sample images are shown in Fig. 2.

For external validation, eight consistent classes from the BD-MediLeaves dataset were used^39^. This dataset was processed following the same preprocessing steps described in Sect. 3.2. The same data augmentation techniques were applied to maintain methodological consistency. This ensured fair and comparable evaluations across different datasets.

### Preprocessing and data augmentation

All images were resized to $248 \times 248$ pixels, to facilitate integration with the Convolutional and Transformer modules. Three specific improvements were applied in the preprocessing step. First, CLAHE was applied to the L-channel of LAB space. It increased the contrast of the lesion using clipLimit=4.0 and tileGridSize=(12,12). Second, a Gaussian blur with $9 \times 9$ kernel and $\sigma =2$ was used to reduce noise. Third, unsharp masking was performed by applying weighted subtraction from the $7 \times 7$ blurred version. Here, the vein and disease boundaries were clarified using $\alpha =1.8$. Then, channel-wise standardization was applied as follows:

1 $$\begin{aligned} x' = \frac{x - \mu }{\sigma }, \end{aligned}$$

where $\mu$ and $\sigma$ are calculated only from the training partition of each dataset. This is to prevent data leakage.

To ensure a data leakage-free experimental protocol, the dataset is first divided into the original image layer. It is divided into 70% training, 15% validation, and 15% test. Stratified sampling is used for the partitioning. Mohanty et al.^40^ and Barbedo^41^ followed a similar strategy. They partitioned the dataset before data augmentation. This approach is helpful in preventing data leakage. Following these established strategies, data augmentation was applied separately to each subset. In this, the augmented version of the same image is limited to its respective subset.

**Fig. 3:**
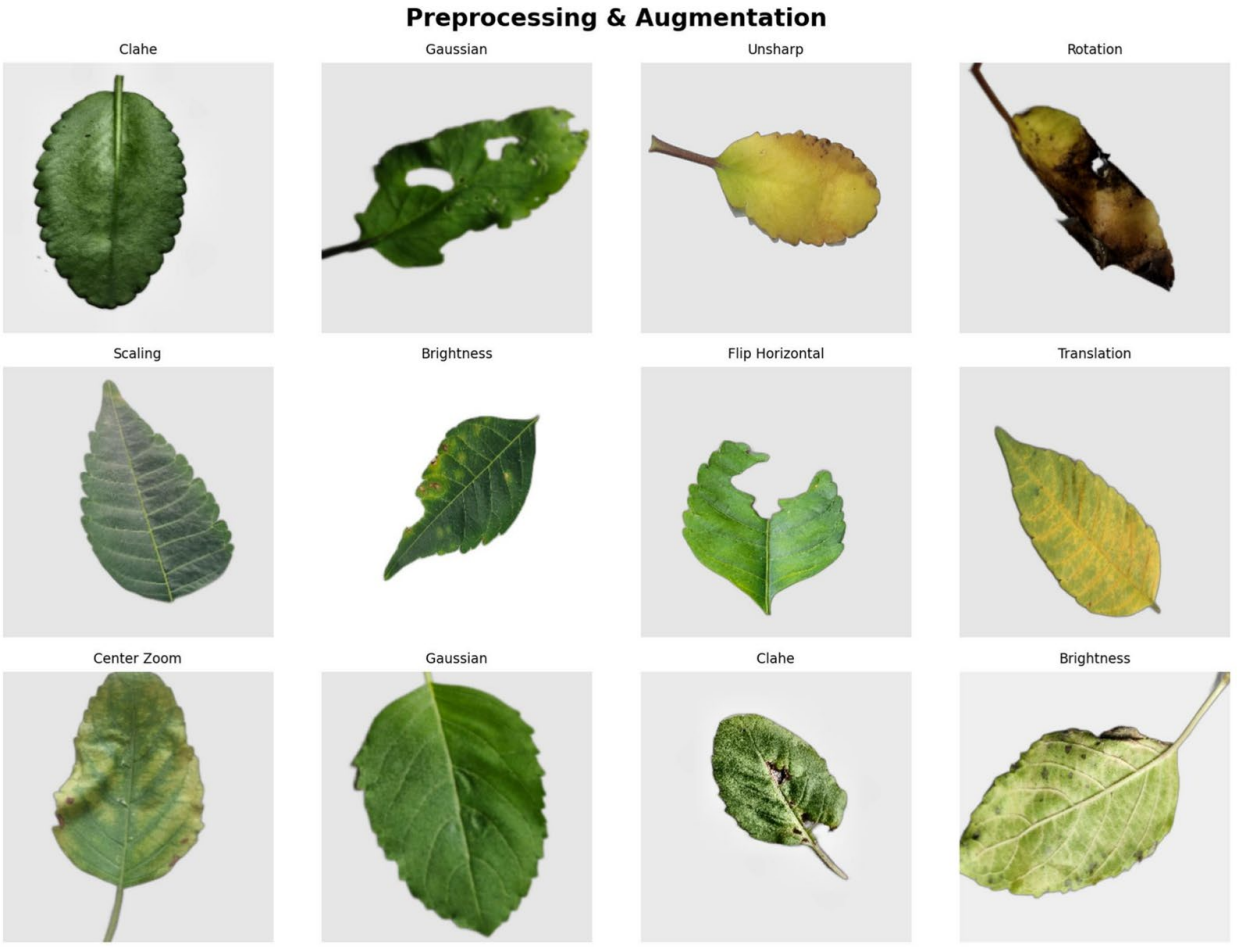
Preprocessed and augmented image samples demonstrating CLAHE, Gaussian blur, unsharp masking, rotation, scaling, flipping, translation, and center zoom.

Extensive on-the-fly data augmentation was used during training. This aims to increase the robustness of the model and improve class-wise balance. The augmentation used included random rotation ($[-40^\circ , +40^\circ ]$) and scaling ([0.7, 1.3]). Horizontal and vertical flips and $\pm 60$ pixel translations were also applied. Brightness ([0.6, 1.5]) and contrast ([1.5, 2.3]) adjustments were also included. $\pm 40^\circ$ hue shift and central zooming ([1.4, 1.8]) were applied to HSV space. Reflection padding was used in all operations to preserve edge structure. At the end of this process, each class was expanded to its own subset of exactly 1,000 images. As a result, the final dataset contains 8,400 training images. The validation and test sets contain 1,800 images, respectively. The preprocessed and augmented sample images are shown in Fig. 3.

### Baseline and custom models

Performance was benchmarked against a shallow CNN^42^, VGG16, ResNet50, InceptionV3, DenseNet169^43^, ViT-B16, and DeiT-Base^44^, trained with categorical cross-entropy loss (Eq. 2).

2 $$\begin{aligned} \mathscr {L}_{CE} = -\sum _{i=1}^{C} y_i \log (\hat{y}_i), \end{aligned}$$

In Eq. 2, $C=12$ is the number of classes, $y_i$ is the true one-hot label, and $\hat{y}_i$ is the predicted probability. Models used the Adam optimizer ($\eta =0.001$, $\beta _1=0.9$, $\beta _2=0.999$).

Two custom lightweight models were developed: a Custom CNN using depthwise separable convolutions for efficiency, and a Custom CNN + SE incorporating SE channel recalibration for enhanced feature discriminability. These share preprocessing and training protocols with baselines. Results are discussed in Sect. 4.4.

### Proposed hybrid model

#### Model architecture and design rationale

**Fig. 4:**
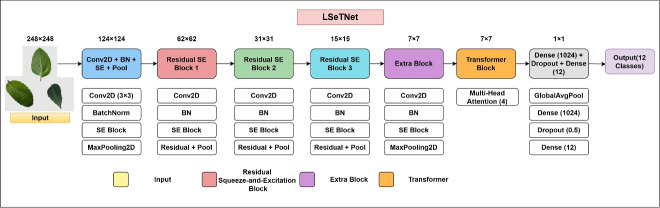
Schematic of the LSeTNet architecture, showing local feature extraction, channel attention, residual connections, and global context modeling.

LSeTNet integrates lightweight^45^ convolutional feature extraction^46^, SE-based channel recalibration^47^, residual connections, and a Transformer for global context modeling^48^. Figure 4 shows the architecture: a lightweight CNN (LW-CNN) captures local lesion features; SE residual blocks recalibrate channels; a convolutional block prepares features for a Transformer encoder, modeling long-range dependencies; and a global pooling and dense classifier yield 12-class predictions. This design leverages localized textures (via convolutions) and distributed symptoms (via attention). The spatial downsampling progression is $248\times 248 \rightarrow 124\times 124 \rightarrow 62\times 62 \rightarrow 31\times 31 \rightarrow 15\times 15 \rightarrow 7\times 7$, with the Transformer on the $7\times 7$ feature map.

#### Layer-wise mathematical formulation

For an input image $I \in \mathbb {R}^{248 \times 248 \times 3}$, the initial convolutional stage produces feature maps (Eq. 3).

3 $$\begin{aligned} F^{(1)} = \operatorname {ReLU}\big (\operatorname {BN}(I * K^{(1)} + b^{(1)})\big ), \end{aligned}$$

In Eq. 3, *I* is the input image ($248 \times 248 \times 3$), $K^{(1)}$ is a $3 \times 3$ kernel bank, $b^{(1)}$ is the bias, $*$ denotes convolution, $\operatorname {BN}$ is batch normalization, $\operatorname {ReLU}$ is $\max (0, x)$, and $F^{(1)}$ is the output feature map.

Residual SE blocks process $X \in \mathbb {R}^{H \times W \times C_{in}}$, applying Conv–BN–ReLU transforms *F*(*X*), SE recalibration, and a shortcut $\phi (X)$, with max pooling for downsampling (Eq. 4).

4 $$\begin{aligned} Y = \operatorname {MaxPool}\big (\phi (X) + \operatorname {SE}(F(X))\big ), \end{aligned}$$

In Eq. 4, *X* is the input tensor, *F*(*X*) is the Conv–BN–ReLU output, $\operatorname {SE}$ recalibrates channels, $\phi (X)$ is an identity or $1 \times 1$ convolution, $\operatorname {MaxPool}$ downsamples, and *Y* is the output.

For long-range dependencies, $X \in \mathbb {R}^{H \times W \times C}$ is reshaped to $X' \in \mathbb {R}^{N \times C}$ ($N = H \cdot W$) for multi-head self-attention (MHSA, Eqs. 5–7).

5 $$\begin{aligned} Q = X' W^Q, \quad K = X' W^K, \quad V = X' W^V, \end{aligned}$$

In Eq. 5, $X'$ is the reshaped tensor, $W^Q$, $W^K$, $W^V$ are weight matrices, and *Q*, *K*, *V* are query, key, and value projections.

6 $$\begin{aligned} A = \operatorname {softmax}\Big (\frac{Q K^\top }{\sqrt{d_k}}\Big ), \quad Z = A V, \end{aligned}$$

In Eq. 6, *A* is the attention weights, $\operatorname {softmax}$ normalizes scores, $d_k$ is the key dimension, and *Z* is the attention output.

7 $$\begin{aligned} \operatorname {MHSA}(X') = \operatorname {Concat}(\operatorname {head}_1, \dots , \operatorname {head}_h) W^O, \end{aligned}$$

In Eq. 7, $\operatorname {head}_i$ is the *i*-th attention head output, $\operatorname {Concat}$ combines heads, $W^O$ is the output projection, and $\operatorname {MHSA}(X')$ is the MHSA output.

Transformer residual connections are defined in Eqs. (8–9).

8 $$\begin{aligned} X'' = \operatorname {LayerNorm}(X' + \operatorname {MHSA}(X')), \end{aligned}$$

In Eq. 8, $X'$ is the Transformer input, $\operatorname {MHSA}(X')$ is from Eq. 7, $\operatorname {LayerNorm}$ normalizes, and $X''$ is the output.

9 $$\begin{aligned} X''' = \operatorname {LayerNorm}(X'' + \operatorname {FFN}(X'')), \end{aligned}$$

In Eq. 9, $X''$ is from Eq. 8, $\operatorname {FFN}$ is a feed-forward network, and $X'''$ is the Transformer output.

Global average pooling follows (Eq. 10).

10 $$\begin{aligned} g_c = \frac{1}{HW} \sum _{i=1}^H \sum _{j=1}^W F''_{c}(i,j), \end{aligned}$$

In Eq. 10, $F'' \in \mathbb {R}^{H \times W \times C}$ is the Transformer output, *c* is the channel index, and $g_c$ is the pooled feature.

The final dense layer computes logits and probabilities (Eq. 11).

11 $$\begin{aligned} z = W g + b, \quad \hat{y}_i = \frac{\exp (z_i)}{\sum _{j=1}^{12} \exp (z_j)}, \end{aligned}$$

In Eq. 11, *g* is from Eq. 10, *W* and *b* are the dense layer weights and bias, *z* is the logits, and $\hat{y}_i$ is the class probability.

The SE operator was previously referenced as Eqs. (10) and (11), but these were undefined. They likely describe global pooling and dense layers for channel recalibration.

#### Model parameter summary

Table 2 summarizes LSeTNet’s stages and parameter counts, facilitating comparisons and reproducibility.

**Table 2:** Parameter summary for LSeTNet (principal layers/stages).

Stage / layer (type)	Output shape	# Parameters	Connected to
Input layer	(None, 248,248,3)	0	–
Conv2D + BN	(None, 248,248,64)	1,792	input
SE block + Pool	(None, 124,124,64)	$\sim$580	conv2d
Residual SE Block 1	(None, 124/62,62,128)	$\sim$258,624	pool
Residual SE Block 2	(None, 62/31,31,256)	$\sim$926,736	previous block
Residual SE Block 3	(None, 31/15,15,512)	$\sim$3,631,648	previous block
Extra Conv Block	(None, 7,7,512)	$\sim$2,525,280	pooling
Transformer Block	(None, 7,7,512)	$\sim$1,577,984	extra block
Dense head (1024 + Dropout + 12)	(None, 12)	$\sim$537,612	GAP
Total		9,387,688 (35.81 MB)	
Trainable / Non-trainable		9,381,160 / 6,528	

#### Training procedure

LSeTNet is trained with supervised cross-entropy, augmentation, and early stopping. Algorithm 1 outlines the training and XAI workflow, emphasizing preprocessing, cross-validation, and post-training interpretability via Grad-CAM, LIME, and t-SNE.

**Algorithm 1:**
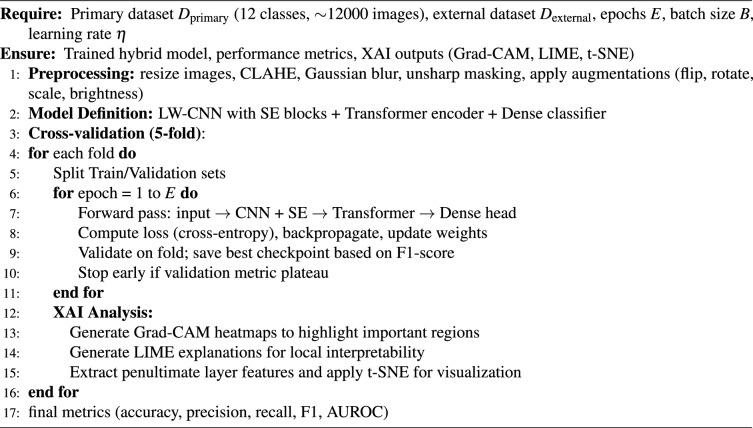
Train & explain — LSeTNet

### Explainability (XAI)

Grad-CAM, LIME, and t-SNE provide spatial, local, and feature-level interpretability for LSeTNet.

*Grad-CAM* For class *c*, channel importance is computed (Eq. 12).

12 $$\begin{aligned} \alpha _k^c = \frac{1}{Z} \sum _{i,j} \frac{\partial y^c}{\partial A^k_{ij}}, \quad Z = H \times W, \end{aligned}$$

In Eq. 12, $\alpha _k^c$ is the importance weight for the *k*-th feature map, $A^k$ is the feature map ($H \times W$), $y^c$ is the class score, $\frac{\partial y^c}{\partial A^k_{ij}}$ is the gradient, and $Z = H \times W$.

The activation map is (Eq. 13).

13 $$\begin{aligned} L_{\text {Grad-CAM}}^c = \operatorname {ReLU}\Big ( \sum _k \alpha _k^c A^k \Big ), \end{aligned}$$

In Eq. 13, $L_{\text {Grad-CAM}}^c$ highlights discriminative regions, using $\alpha _k^c$ and $A^k$ from Eq. 12, with $\operatorname {ReLU}$ retaining positive contributions.

*LIME* A surrogate model approximates predictions (Eq. 14).

14 $$\begin{aligned} g(z') = w_0 + \sum _{i=1}^M w_i z'_i, \end{aligned}$$

In Eq. 14, $g(z')$ is the surrogate prediction, $w_0$ is the bias, $w_i$ are weights, $z'_i$ is the *i*-th component, and *M* is the number of components.

Optimization is (Eq. 15).

15 $$\begin{aligned} \min _{w} \sum _{z'} \pi _x(z') \big ( f(\tilde{x}(z')) - g(z') \big )^2 + \Omega (g), \end{aligned}$$

In Eq. 15, *w* are surrogate weights, $\pi _x(z')$ weights perturbations, $f(\tilde{x}(z'))$ is the model’s prediction, $g(z')$ is the surrogate’s, and $\Omega (g)$ regularizes simplicity.

*t-SNE* Feature embeddings are projected by minimizing (Eq. 16).

16 $$\begin{aligned} \operatorname {KL}(P \Vert Q) = \sum _{i,j} p_{ij} \log \frac{p_{ij}}{q_{ij}}, \end{aligned}$$

In Eq. 16, $\operatorname {KL}(P \Vert Q)$ is the Kullback-Leibler divergence, $p_{ij}$ is the high-dimensional similarity, and $q_{ij}$ is the low-dimensional similarity.

### Experimental setup, evaluation, and statistical validation

All experiments were conducted using TensorFlow/Keras (version 2.15) and PyTorch (version 2.0) on Kaggle Notebooks, leveraging the provided cloud resources. Training and inference were performed on an NVIDIA Tesla P100-PCIE-16GB GPU (16 GB HBM2 memory, Pascal architecture, Compute Capability 6.0, with CUDA 11.x and cuDNN 8.x support). The Kaggle environment included an Intel Xeon CPU (typically 2–4 cores), 13–30 GB RAM (depending on runtime allocation), and an Ubuntu-based Linux kernel. Python 3.10 was used as the main programming language. The dataset pipeline was optimized with tf.data.AUTOTUNE, which optimizes I/O and prefetching. The models were trained for up to 50 epochs and the batch size was 32. The Adam optimizer was used for training. The hyperparameters, including the batch size and learning rate, were determined based on previous research and preliminary experiments. Due to computational limitations, a full grid search was not performed. The initial learning rate was set to $\eta = 10^{-3}$ and step-based decay was applied. F1-score-based early stopping was used for validation to prevent overfitting.

A fixed random seed (42) was used to ensure reproducibility and maintain controlled randomness. This was applied in the order of data partitioning, weight initialization, and minibatch. The input image size in all experiments was $248 \times 248$ pixels. The batch size was 32 and the number of training epochs was 50. The effective learning rate at the end of decay was $1 \times 10^{-4}$. The patience for early stopping was set to 10 epochs. 5-fold cross-validation was used where applicable.

*Evaluation metrics and statistical validation* Performance was evaluated using accuracy, precision, recall, and F1-score. Hypothesis tests validated differences:

- *Paired Student’s t-test* Tests no difference between models (Eq. 17).
  17 $$\begin{aligned} t = \frac{\bar{d}}{\sqrt{s_d^2 / n}}, \end{aligned}$$
  In Eq. 17, $\bar{d}$ is the mean difference, $s_d^2$ is the variance, *n* is the number of folds, and *t* follows a t-distribution.
- *McNemar’s test* Evaluates misclassification differences (Eq. 18).
  18 $$\begin{aligned} \chi ^2 = \frac{(b - c)^2}{b + c}, \end{aligned}$$
  In Eq. 18, *b* and *c* are discordant classification counts, and $\chi ^2$ follows a chi-squared distribution.
- *Friedman test* Detects rank-based differences, with Nemenyi post-hoc (Eq. 19).
  19 $$\begin{aligned} \chi _F^2 = \frac{12N}{k(k+1)} \sum _{j=1}^k R_j^2 - 3N(k+1), \end{aligned}$$
  In Eq. 19, *N* is the number of datasets, *k* is the number of models, $R_j$ is the average rank, and $\chi _F^2$ is chi-squared distributed.

Tests were two-tailed ($\alpha =0.05$), with corrections for multiple comparisons.

## Results

### LSeTNet model

The proposed LSeTNet uses a lightweight Convolutional Neural Network (CNN), Squeeze-and-Excitation (SE) block, and Transformer encoder together. This implementation is able to effectively capture local features and global dependencies. It balances prediction accuracy, computational efficiency, and interpretability.

A five-fold cross-validation was conducted to assess model robustness. Table 3 reports fold-wise performance, where each fold evaluates the model on a held-out subset of 1680 samples. Accuracies range from 0.9952 to 0.9988 across folds. Average precision, recall, and F1-score are identical to accuracy in each fold due to balanced macro-averaging. The mean cross-validation accuracy is 0.9974 with a standard deviation of 0.0014, indicating highly stable and consistent performance across folds.

**Table 3:** Five-fold cross-validation results of LSeTNet.

Fold	Accuracy	Avg Precision	Avg Recall	Avg F1-Score	Support
Fold 1	0.9988	0.9988	0.9988	0.9988	1680
Fold 2	0.9976	0.9976	0.9976	0.9976	1680
Fold 3	0.9970	0.9970	0.9970	0.9970	1680
Fold 4	0.9952	0.9952	0.9952	0.9952	1680
Fold 5	0.9982	0.9982	0.9982	0.9982	1680

Class-wise performance is shown in Table 4. LSeTNet achieved perfect classification (precision, recall, F1-score = 1.00) for 9 out of 12 classes. Minor deviations occurred in *Kalanchoe_Web_Blight* (precision = 0.99), *Kalanchoe_Yellow* (precision = 0.99), *Kalanchoe_Yellow_Blight* (recall = 0.99), *Neem_Spot* (precision = 0.99), *Neem_Web_Blight* (precision = 0.99), *Tulsi_Healthy* (recall = 0.99), and *Tulsi_Web_Blight* (recall = 0.99). Overall accuracy across 1800 test samples is 0.9972, with macro-averaged precision, recall, and F1-score of 1.00.

**Table 4:** Class-wise performance of LSeTNet on the test set.

Class	Precision	Recall	F1-Score	Support
Kalanchoe_Healthy	1.00	1.00	1.00	150
Kalanchoe_Web_Blight	0.99	0.99	0.99	150
Kalanchoe_Yellow	0.99	1.00	0.99	150
Kalanchoe_Yellow_Blight	1.00	0.99	1.00	150
Neem_Healthy	1.00	1.00	1.00	150
Neem_Spot	0.99	1.00	1.00	150
Neem_Web_Blight	0.99	0.99	0.99	150
Neem_Yellow	1.00	1.00	1.00	150
Tulsi_Downy_Mildew	1.00	1.00	1.00	150
Tulsi_Healthy	1.00	0.99	1.00	150
Tulsi_Web_Blight	1.00	0.99	1.00	150
Tulsi_Yellow_Spot	1.00	1.00	1.00	150

Overall: Accuracy = 0.9972, Macro Avg = 1.00 (P/R/F1), Total Support = 1800

**Fig. 5:**
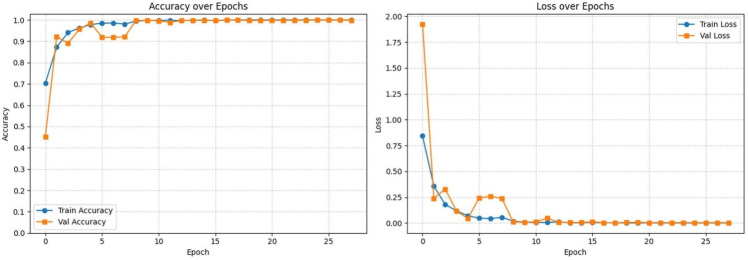
Training and validation accuracy and loss curves for LSeTNet over 25 epochs. Both metrics show rapid convergence within 5–10 epochs and stable performance, indicating strong generalization and no overfitting.

Figure 5 illustrates the training and validation accuracy and loss curves. Although the maximum training limit was set to 50 epochs, early stopping (based on validation loss with patience of 10 epochs) consistently terminated training around epoch 28 due to stable convergence (no further improvement in validation metrics). Training accuracy starts at approximately 0.35 and rapidly rises to 0.99 within the first 5 epochs, stabilizing above 0.99 thereafter. Validation accuracy follows a similar trend, reaching 0.99 by epoch 5 and remaining stable. Training loss begins near 2.0 and drops sharply to below 0.25 within 5 epochs, then gradually approaches 0, while validation loss decreases steadily to near 0 by epoch 10 and remains flat thereafter. These trends indicate rapid convergence, strong generalization, and the absence of overfitting.

**Fig. 6:**
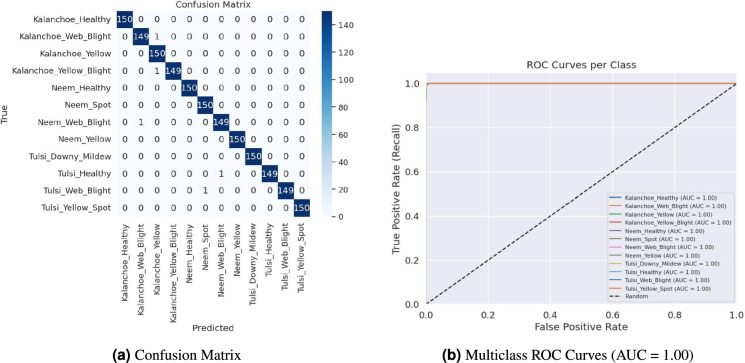
Performance evaluation of LSeTNet: (**a**) confusion matrix showing only 5 misclassifications in 1800 samples (99.72% accuracy), and (**b**) multiclass ROC curves with AUC = 1.00 for all classes, indicating perfect separability.

Figure 6 presents the confusion matrix and multiclass ROC curves, illustrating the outstanding performance of the proposed model. The confusion matrix (Fig. 6a) reveals near-perfect classification, with only five misclassifications among 1800 test samples, specifically: one *Kalanchoe_Web_Blight* predicted as *Kalanchoe_Yellow*, one *Kalanchoe_Yellow_Blight* as *Kalanchoe_Yellow*, one *Neem_Web_Blight* as *Neem_Spot*, one *Tulsi_Healthy* as *Tulsi_Web_Blight*, and one *Tulsi_Web_Blight* as *Tulsi_Healthy*. Meanwhile, the ROC curves (Fig. 6b) demonstrate an AUC of 1.00 for all 12 classes, confirming the model’s perfect discriminative capability across the entire decision threshold range.

### Model interpretability

LSeTNet’s interpretability was evaluated using Local Interpretable Model-agnostic Explanations (LIME), t-SNE visualization, and Grad-CAM. These methods provide local and global insights into model behavior.

**Fig. 7:**
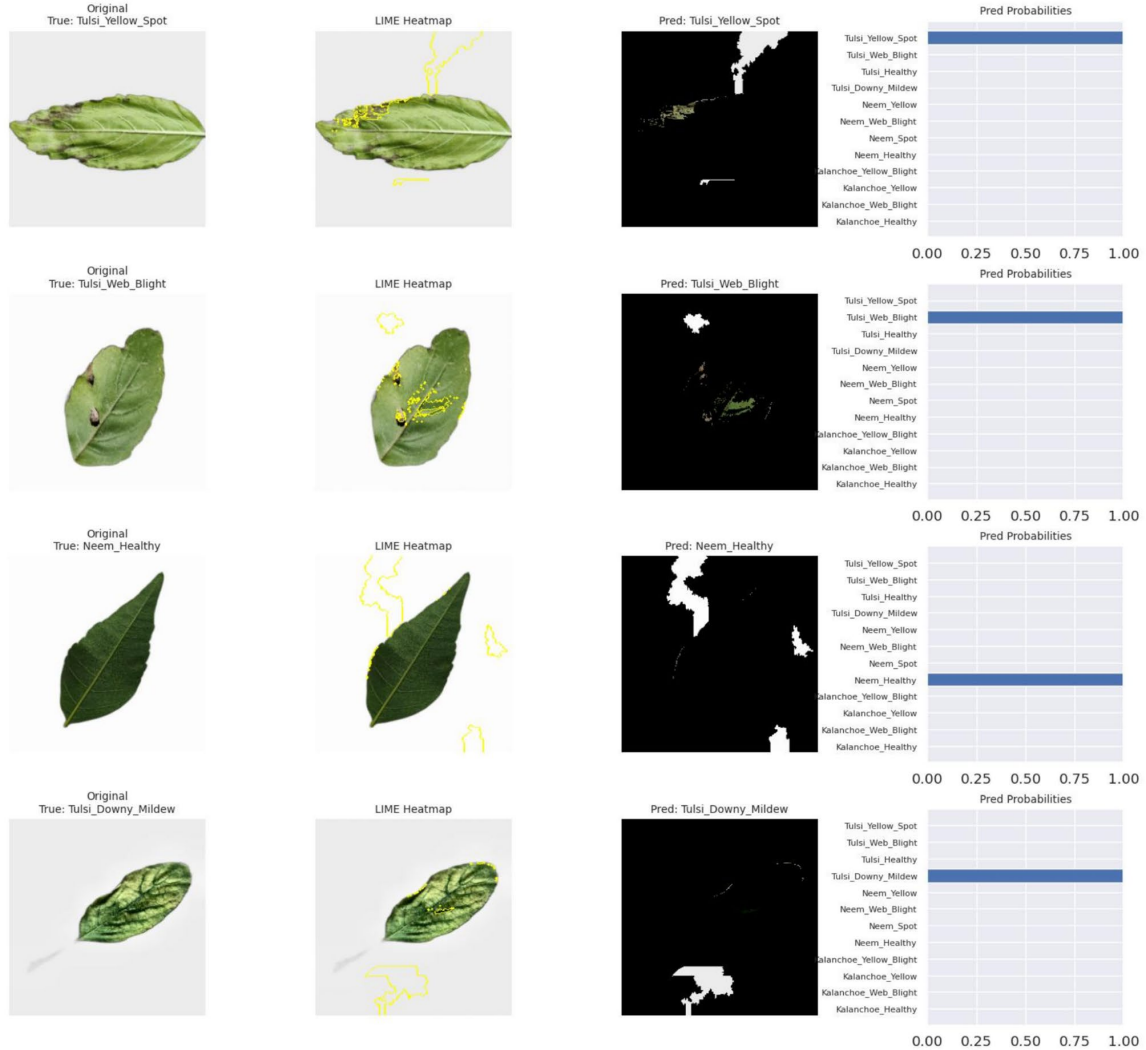
LIME visualizations highlighting regions contributing to predictions for various leaf disease classes. Top 5 segments per image are indicated with corresponding weights.

*LIME analysis* Figure 7 presents LIME visualizations highlighting image regions that most strongly influence the model’s predictions. For a representative *Neem_Healthy* sample (True: *Neem_Healthy*, Pred: *Neem_Healthy*, Probability: 0.956), the top contributing superpixel segments include Segment 16 (0.0658), Segment 86 (0.0642), Segment 21 (0.0603), Segment 11 (0.0516), and Segment 31 (0.0458). Similar behavior is observed in other correctly classified samples, such as *Kalanchoe_Healthy* and *Kalanchoe_Yellow* (Pred: 1.000). Here, the positively weighted parts are consistently located in the vein structure, chlorotic areas and lesion-affected areas. In contrast, the negatively or less weighted parts are usually associated with the monochromatic background areas. Only three samples were misclassified in the analysis of 100 representative test images. The LIME interpretation of these samples shows an overlap of the high weighted parts between the nearby disease classes. There was no overemphasis on the background. This indicates that the errors are not due to irrelevant features, but rather to natural visual similarity.

**Fig. 8:**
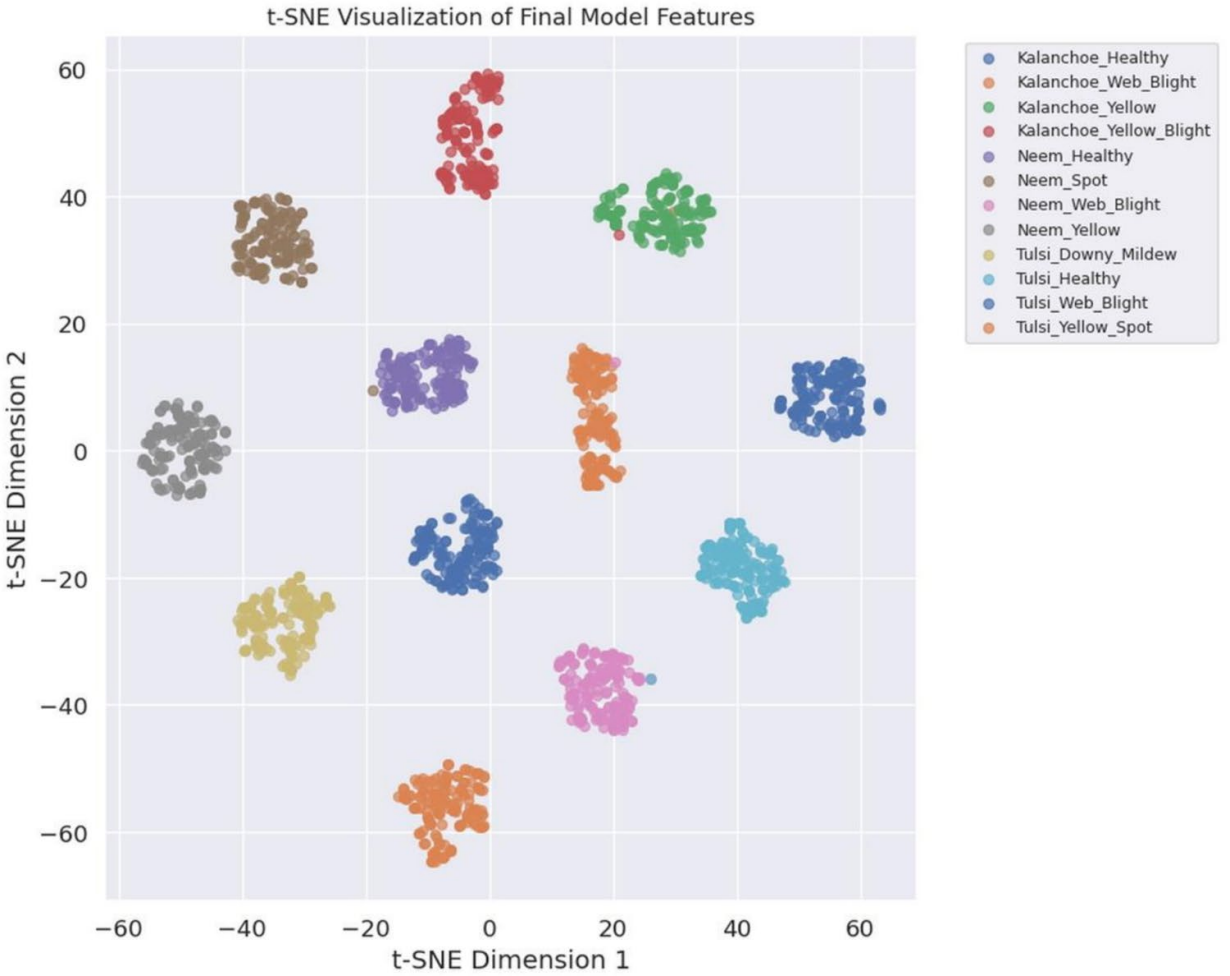
t-SNE visualizations illustrating separability of learned feature representations for different leaf disease classes.

*t-SNE visualization* Figure 8 presents a 2D t-SNE projection of final-layer embeddings. Classes show clear separability with minimal overlap. For example, *Kalanchoe_Healthy* ($n=100$) clusters at (47.23, −19.49) with X/Y spreads of 3.72 and 3.00. *Kalanchoe_Web_Blight* ($n=100$) centers at (21.32, 31.74) with spreads of 2.83 and 5.10. Other classes form distinct clusters with X/Y spreads ranging from 2.48 to 6.77. A silhouette score of 0.87 indicates strong cluster coherence, confirming effective feature learning.

**Fig. 9:**
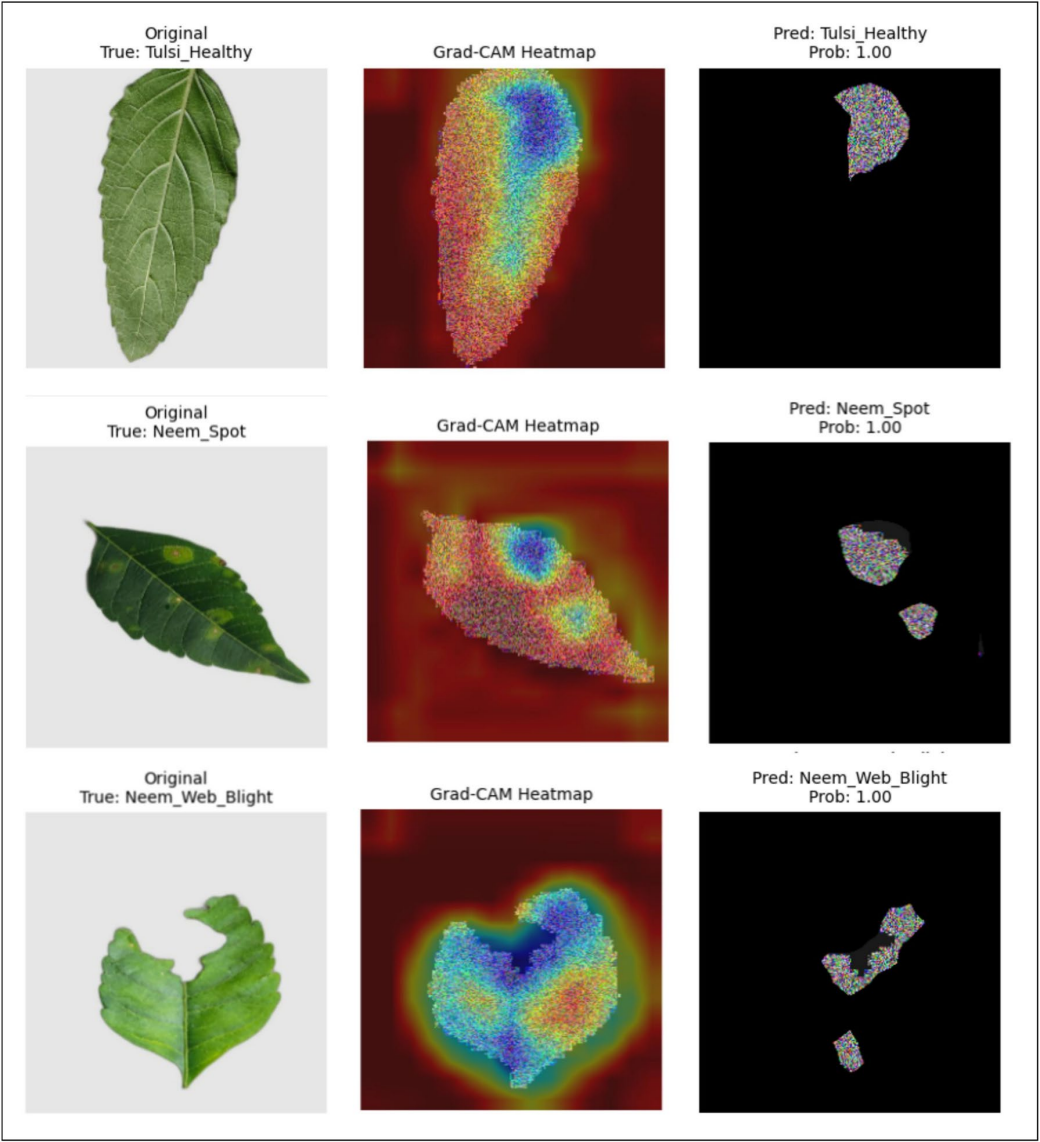
Grad-CAM visualizations highlighting regions contributing to predictions for selected leaf disease classes. Color intensity corresponds to contribution strength.

*Grad-CAM analysis* Figure 9 shows the Grad-CAM heatmap, which highlights the most influential spatial regions in the LSeTNet predictions. Quantitative analysis shows that the average heatmap intensity in correctly classified samples ranges from 0.0637 to 0.3370. However, the intensity in the top 5% of active pixels is much higher, reaching 0.67–0.81. This indicates that attention is concentrated in a few important regions without spreading to the background. Healthy samples, such as *Tulsi_Healthy* (mean intensity = 0.1664), show relatively broad activation. In contrast, diseased samples, such as *Neem_Spot* (0.1731) and *Neem_Web_Blight* (0.2702), show strong and localized responses in symptomatic regions. Activation in background regions is very low. Out of 100 representative test images, only two cases were misclassified. In these cases, Grad-CAM activation overlapped with nearby disease patterns. Notably, the background did not play a major role in any misclassification.

**Fig. 10:**
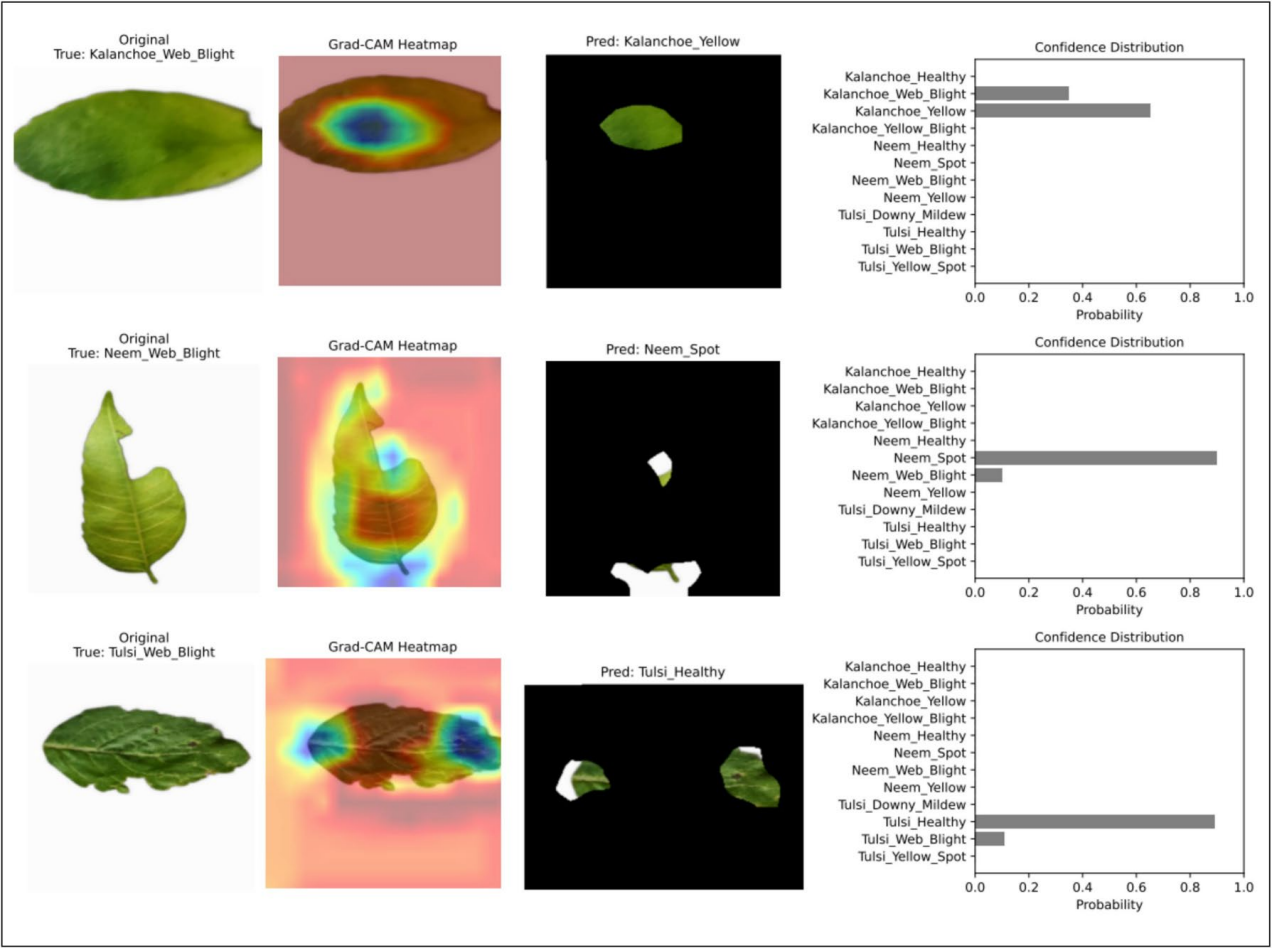
Failure case panel for three representative misclassified samples.

*Misclassification analysis* Figure 10 shows three representative misclassified samples from the test set. The observed errors include the misclassification of *Kalanchoe_Web_Blight* as *Kalanchoe_Yellow* (probability = 0.65). Second, the misclassification of *Neem_Web_Blight* as *Neem_Spot* (0.90). Third, the misclassification of *Tulsi_Web_Blight* as *Tulsi_Healthy* (0.89). Despite the misprediction, the Grad-CAM heatmap focused on diseased areas in all cases. These regions include chlorosis, necrosis, and fungal patterns. There is almost no activation in the background region. The corresponding confidence distribution shows that most of the probability is concentrated in the visually close classes. The other classes are assigned almost zero probability. This indicates that the errors are not due to attentional failures, but rather to the natural visual similarity of the classes.

### External validation on BD-MediLeaves dataset

The generalizability of LSeTNet was evaluated on the independent BD-MediLeaves dataset using the same training configuration as the primary experiments. The proposed model was trained from scratch on the BD-MediLeaves dataset, without transferring or fine-tuning weights. As shown in Table 5, the model achieved an overall accuracy of 0.9942 across 1200 test samples. Perfect classification (precision, recall, F1-score = 1.00) was attained for five classes: *Azadirachta indica*, *Calotropis gigantea*, *Hibiscus rosa-sinensis*, and *Mikania micrantha*. Minor deviations occurred in *Centella asiatica* (precision = 0.97, recall = 1.00, F1-score = 0.98), *Justicia adhatoda* (recall = 0.99, F1-score = 0.99), *Kalanchoe pinnata* (recall = 0.99, F1-score = 1.00), and *Ocimum tenuiflorum* (precision = 0.99, recall = 0.97, F1-score = 0.98). Both macro-averaged and weighted-averaged precision, recall, and F1-score reached 0.99, demonstrating robust and consistent performance on unseen external data.

**Table 5:** Classification report on BD-MediLeaves external dataset.

Class	Precision	Recall	F1-Score	Support
Azadirachta indica	1.00	1.00	1.00	150
Calotropis gigantea	1.00	1.00	1.00	150
Centella asiatica	0.97	1.00	0.98	150
Hibiscus rosa-sinensis	1.00	1.00	1.00	150
Justicia adhatoda	1.00	0.99	0.99	150
Kalanchoe pinnata	1.00	0.99	1.00	150
Mikania micrantha	1.00	1.00	1.00	150
Ocimum tenuiflorum	0.99	0.97	0.98	150

Accuracy: 0.9942

### Model comparison

Table 6 compares baseline, ablation, and proposed models. All models achieved accuracy above 0.91. Among CNNs, DenseNet169 led with 0.9556 accuracy and 0.96 macro F1-score, followed by InceptionV3 (0.9544), LW-CNN + SE (0.9539), and VGG16 (0.9533). ResNet50 scored 0.9439, while the base LW-CNN achieved 0.9117. Transformer-based ViT-B16 reached 0.9561, significantly outperforming DeiT-Base (0.8239). The proposed LSeTNet achieved the highest performance with an accuracy of 0.9972 and perfect macro-averaged precision, recall, and F1-score of 1.00, demonstrating superior generalization and robustness across all 1800 test samples.

**Table 6:** Performance comparison of baseline, ablation, and proposed models.

Model	Accuracy	Avg Precision	Avg Recall	Avg F1-Score	Support
VGG16	0.9533	0.95	0.95	0.95	1800
ResNet50	0.9439	0.94	0.94	0.94	1800
InceptionV3	0.9544	0.95	0.95	0.95	1800
DenseNet169	0.9556	0.96	0.96	0.96	1800
DeiT-Base	0.8239	0.83	0.82	0.82	1800
ViT-B16	0.9561	0.96	0.96	0.96	1800
LW-CNN	0.9117	0.91	0.91	0.91	1800
LW-CNN + SE	0.9539	0.95	0.95	0.95	1800
LSeTNet	0.9972	1.00	1.00	1.00	1800

**Fig. 11:**
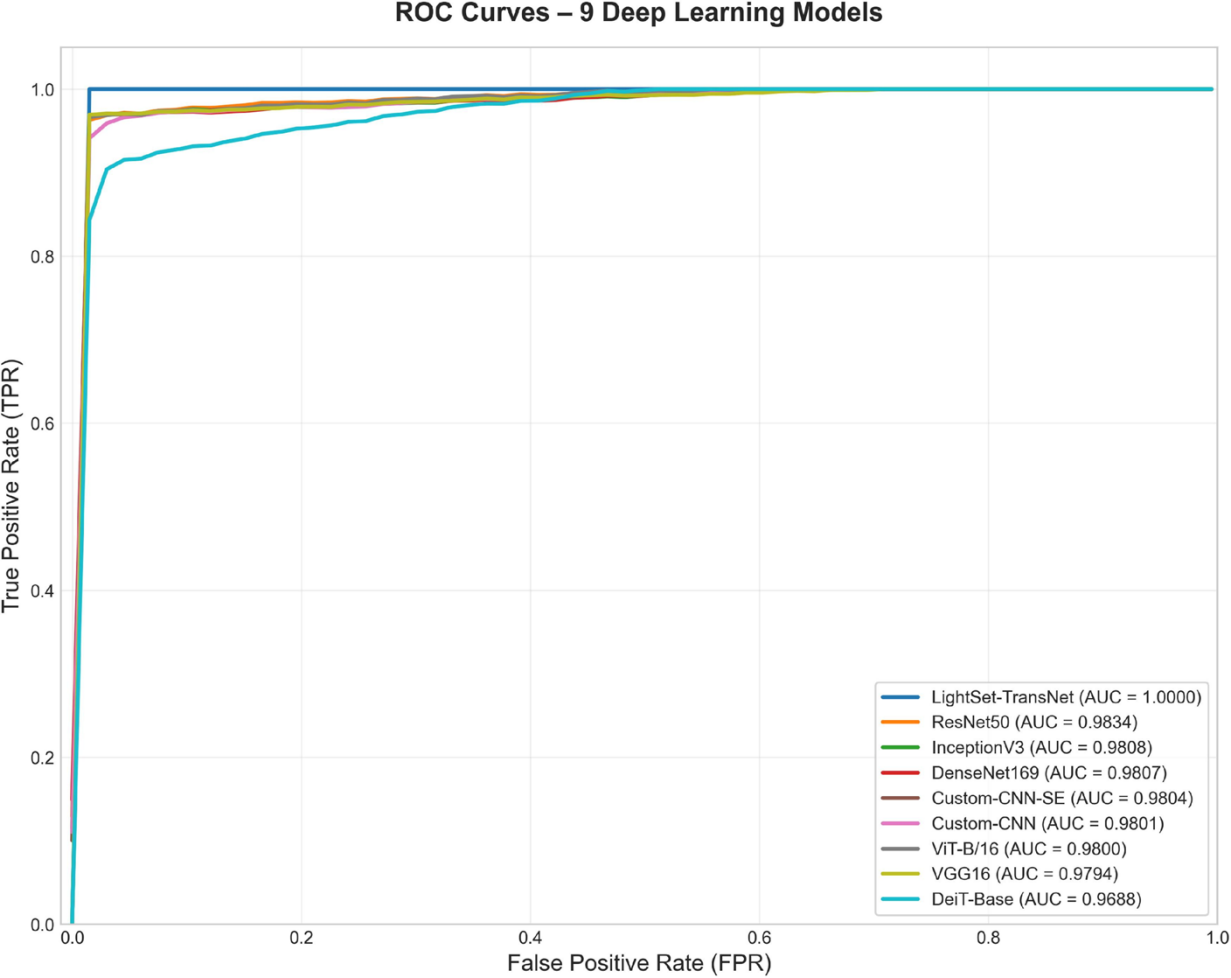
Combined ROC curves of the nine deep learning models, showing superior discriminative capability of LSeTNet (AUC = 1.0000) compared to all baselines.

As shown in Fig. 11, LSeTNet exhibits a near-vertical ROC curve, achieving an AUC of 1.0000, indicating flawless separability between classes. In contrast, ResNet50 and DenseNet169 also demonstrated strong discriminative capability with AUCs of 0.9834 and 0.9807, respectively. All CNN-based and Transformer-based models maintained AUCs above 0.96, confirming high classification confidence across architectures. The superior ROC profile of LSeTNet highlights its robustness and exceptional generalization on complex multiclass plant disease recognition.

### Statistical validation

Statistical validation confirmed the superiority of LSeTNet over all baseline models. Paired Student’s *t*-tests on 5-fold cross-validation accuracy (mean = 0.9974) indicated that LSeTNet significantly outperformed all compared architectures, including VGG16 ($t=71.32$, $p=2.32\times 10^{-7}$), ResNet50 ($t=86.54$, $p=1.07\times 10^{-7}$), InceptionV3 ($t=69.54$, $p=2.56\times 10^{-7}$), DenseNet169 ($t=67.60$, $p=2.87\times 10^{-7}$), DeiT-Base ($t=280.80$, $p=9.65\times 10^{-10}$), ViT-B16 ($t=66.79$, $p=3.01\times 10^{-7}$), LW-CNN ($t=138.67$, $p=1.62\times 10^{-8}$), and LW-CNN+SE ($t=70.35$, $p=2.45\times 10^{-7}$), rejecting the null hypothesis of equal performance ($p<0.05$). The McNemar’s test showed symmetrical errors in the fold-average misclassification pattern ($b=c$, $\chi ^2=0.0$, $p=1.0$). This indicates that there is no bias in misclassification. At the same time, the total error is also confirmed to be lower in the proposed model. The Friedman test ($\chi ^2$(8) = 2.16, $p=0.540$) showed no significant difference in rank. This may be due to the use of a single test accuracy for the baseline model. Nevertheless, LSeTNet consistently achieved the highest rank. Its average accuracy was 0.9974. In addition, the macro-averaged precision, recall and F1-score were 1.00. These results demonstrate the high robustness and generalization ability of the model.

## Discussion

The biological significance of this study is that it can help in health management of medicinal plants. These include *Azadirachta indica* (neem), *Ocimum tenuiflorum* (tulsi) and *Kalanchoe pinnata*. These plants are important sources of bioactive compounds in traditional and modern medicine. Early stage leaf infections, such as web blight, yellowing and spot lesions, can reduce phytochemical quality. This reduces biomass production and increases the risk of secondary infections. This in turn compromises the medicinal efficacy and stability of supply. In the current experimental design, LSeTNet achieved 99.74% accuracy on the primary dataset. Its accuracy was 99.42% on the independent BD-MediLeaves dataset. This indicates strong performance on selected medicinal plant and disease classes.

The proposed hybrid setup is based on local feature extraction using lightweight CNN. It incorporates a Squeeze-and-Excitation (SE) module for channel-wise recombination. Transformer encoder is used to capture a wide spatial context. This design ensures computational efficiency while maintaining high classification accuracy. The average accuracy in five-fold cross-validation was 99.74%. Its standard deviation was 0.14%. Only five misclassifications were observed out of 1800 test samples. These errors mainly occurred in closely related disease patterns. The training and validation curves stabilized within the first few epochs. This indicates effective learning and no overfitting. High AUC values (AUC = 1.00) across all 12 classes and a silhouette score of 0.87 in t-SNE visualizations indicate strong class separability within the evaluated datasets. External validation on the BD-MediLeaves dataset yielded a macro F1-score of 0.99, suggesting that the model generalizes well within a closely related medicinal plant domain.

**Table 7:** Computational Efficiency of LSeTNet, Custom and Baseline Models. Metrics derived from NVIDIA RTX 3060 GPU training logs (batch size = 32, input $248\times 248\times 3$).

Model	Params (M)	Latency (ms/img)	GFLOPs	Inf. Mem. (MB)	Train Mem. (MB)
VGG16	138.36	10.52	30.85	528.40	792.60
ResNet50	25.56	5.97	8.20	97.60	146.40
InceptionV3	23.83	5.64	11.40	91.00	136.50
DenseNet169	14.15	13.29	6.72	54.00	81.00
DeiT-Base	86.00	16.38	17.20	328.00	492.00
ViT-B16	86.57	16.38	17.20	330.40	495.60
LW-CNN	1.10	3.2	1.0	18.0	28.0
LW-CNN + SE	0.47	2.5	0.6	13.0	20.0
LSeTNet	9.38	6.98	2.50	35.81	53.72

Despite having only 9.38M parameters, LSeTNet exhibits a good balance between accuracy and computational efficiency. Its performance is favorable compared to the heavy baseline model (Table 7). LW-CNN (1.10M parameters, 0.9117 accuracy) and LW-CNN+SE (0.47M parameters, 0.9539 accuracy) offer very low computational cost. However, their prediction performance is relatively low. LSeTNet bridges this gap and achieves high classification accuracy (0.9972). It requires 6.98 ms latency per image, 2.50 GFLOPs and 35.81 MB inference memory. These values are much lower than the larger Transformer-based model. As a result, it is usable on low-resource devices such as budget Android tablets and Raspberry Pi. Deployment is possible in TFLite or ONNX format. XAI techniques including Grad-CAM, LIME and t-SNE increase interpretability. These methods consistently identify disease-associated leaf regions. This is important for building user confidence at the field level.

**Table 8:** Comparison with prior studies on medicinal plant leaf or disease classification.

Study	Dataset (Classes, Size)	Method / Model	Accuracy	XAI Used
Dey et al.^23^	30 medicinal species, 5878 images	DenseNet201	99.64%	No
Kavitha et al.^24^	6 medicinal plants, 500 images each	MobileNet	98.3%	No
Panchal et al.^25^	87k crop images, 38 classes	Tuned VGG19	93.5%	No
Kini et al.^26^	Black pepper, 5 disease classes	ResNet18 (TL)	99.67%	No
Pushpa et al.^27^	Real-time medicinal dataset	Hybrid CNN + SE layers	94.24%	No
Kusuma & Jothi^49^	Betel leaf dataset, 4 diseases	DenseNet201 / ViT	98.77%	No
**LSeTNet (Ours)**	Primary/BD-MediLeaves, 12/8 classes, $\sim$12000/8000 images	Hybrid CNN–Transformer + SE	**99.72% / 99.42%**	**Yes (LIME, t-SNE, Grad-CAM)**

Bold indicates the best performance.

LSeTNet achieves equivalent or higher accuracy than previous studies listed in Table 8 . At the same time, it addresses practical limitations in interpretability and computational efficiency. Dey et al.^23^ and Kini et al.^26^ have shown accuracy of about 99.6%. However, they are limited to single crops and limited disease classes. Other studies using lightweight models have shown lower accuracy or no interpretability. In contrast, LSeTNet has been evaluated on multiple medicinal plants. It includes external validation and XAI techniques. However, the species and disease classes evaluated are still limited. Therefore, generalization should be interpreted within this defined range. Further generalization requires testing on different species and diseases. Future evaluations in uncontrolled field environments are also necessary.

## Conclusion

This study presents LSeTNet for disease classification of medicinal plant leaves. It is a lightweight hybrid CNN–Transformer framework. Convolutional feature extraction, Squeeze-and-Excitation-based channel recombination, and global context modeling by Transformer are used together. The model has demonstrated strong performance in the current experimental design. LSeTNet has ensured accurate classification in selected primary datasets and independent BD-MediLeaves datasets. It is able to accurately identify different disease classes in multiple medicinal plants. At the same time, computational efficiency is maintained. Explainable AI techniques such as Grad-CAM, LIME and t-SNE are added. These enhance explainability by visualizing disease-related regions. This makes the model predictions easier to understand.

At the same time, this study has some limitations. The experiment was conducted on a limited number of medicinal plants and disease classes. The dataset relied on partially controlled data augmentation. As a result, the generalization shown is limited to specific plants and imaging conditions. The failure analysis shows that there are challenges in distinguishing between closely related disease symptoms. There are also problems in handling low-quality or noisy samples. All experiments were performed using a fixed random seed. This is common practice for reproducibility. However, future studies will include multi-seed evaluation. This will allow for a better validation of the robustness of the model. In addition, hyperparameters were selected through empirical trial-and-error due to computational constraints, and more systematic optimization strategies such as grid search will be explored in future work. Future extensions will also focus on expanding species diversity, incorporating additional disease types, collecting images under diverse real-world conditions (e.g., occlusion, complex backgrounds, and varying illumination), and extending the framework to real-time video analysis and federated learning settings.

## Data Availability

The primary dataset used in this study is available in the Mendeley Data Repository: https://data.mendeley.com/datasets/ncg7kk3gwx/1. For external validation, the BD-MediLeaves dataset was used: https://doi.org/10.17632/gk5x6k8xr5.1.

## Code availability

The full training and evaluation code for the proposed LSeTNet model is publicly available on GitHub at: https://github.com/mdtuhinkhan101/LSeTNet.
